# Mechanisms behind Retinal Ganglion Cell Loss in Diabetes and Therapeutic Approach

**DOI:** 10.3390/ijms21072351

**Published:** 2020-03-28

**Authors:** María Constanza Potilinski, Valeria Lorenc, Sofía Perisset, Juan Eduardo Gallo

**Affiliations:** 1 Instituto de Investigaciones en Medicina Traslacional (IIMT), Facultad de Ciencias Biomedicas, Universidad Austral-CONICET, Av. J.D. Perón 1500, 1629 Pilar, Buenos Aires, Argentina; constanza.potilinski@gmail.com (M.C.P.); valelorenc@yahoo.com.ar (V.L.); sofiaperisset@gmail.com (S.P.); 2 Departamento de Oftalmologia, Hospital Universitario Austral, Av. Juan Perón 1500, 1629 Pilar, Buenos Aires, Argentina

**Keywords:** Retinal Ganglion Cells, Diabetic Retinopathy, Signaling pathway, Inflammation, Alpha-1 antitrypsin

## Abstract

Diabetes produces several changes in the body triggered by high glycemia. Some of these changes include altered metabolism, structural changes in blood vessels and chronic inflammation. The eye and particularly the retinal ganglion cells (RGCs) are not spared, and the changes eventually lead to cell loss and visual function impairment. Understanding the mechanisms resulting in RGC damage and loss from diabetic retinopathy is essential to find an effective treatment. This review focuses mainly on the signaling pathways and molecules involved in RGC loss and the potential therapeutic approaches for the prevention of this cell death. Throughout the manuscript it became evident that multiple factors of different kind are responsible for RGC damage. This shows that new therapeutic agents targeting several factors at the same time are needed. Alpha-1 antitrypsin as an anti-inflammatory agent may become a suitable option for the treatment of RGC loss because of its beneficial interaction with several signaling pathways involved in RGC injury and inflammation. In conclusion, alpha-1 antitrypsin may become a potential therapeutic agent for the treatment of RGC loss and processes behind diabetic retinopathy.

## 1. Introduction

Perception of the environment depends on senses, where vision is one of the most complex due to all cell types involved coordinately to form a visual image. Retinal ganglion cells (RGCs) are the last cells involved in light transduction in the retina, they integrate signals encoding specific constituents of an image and transport them to the brain [1,2,3]. In addition, they are involved in transmission of additional light-independent information like cell metabolism state, intraocular pressure or temperature of the eye [4]. Consequently, the damage to these cells results in vision loss.

Diabetic retinopathy is a neuro-vascular disease, one of the leading causes of severe vision loss. Among diabetics the prevalence of retinopathy is approximately 35% [5]. In advanced stages, angiogenesis, proliferative vitreous retinopathy (PVR) and eventually retinal detachment can be observed as well [6]. The main vascular changes produced in diabetic retinopathy are originated by chronic hyperglycemia [7]. At present, there is no preventive treatment for diabetic retinopathy that can be used in the early stages of the disease [8,9,10].

RGCs are damaged in diabetic retinopathy, producing cell function impairment and their subsequent loss [11]. Contrary to expectations, the damage produced on these cells occurs in the early stages of diabetic retinopathy, before the onset of an evident vascular damage [12,13,14,15]. Among the contributors to this pathology that affect RGC viability are hypoxia [16], inflammation [17], oxidative stress [18] and diverse protein pathways involved in these processes, as well as in normal function and metabolism of RGCs.

Available animal models of diabetic retinopathy until now could be classified into genetic animal models and induced animal models. Within the genetic mouse models, the more commonly used are Ins2^Akita^, non-obese diabetic (NOD), db/db (Lepr^db^), Kimba and Akimba. Induced animal models can be developed through five methods: removal of the pancreas, administration of the drug alloxan, administration of the drug streptozotocin (STZ), high-galactose diets, and laser or chemical damage to the eye. In mouse only alloxan, STZ and high-galactose diets are used [19].

The loss of RGC has been observed as characteristic of many of these animal models including Akita, NOD, db/db within the genetic mouse models; and the STZ mouse model. But there are some discrepancies in the occurrence of RGC loss probably due to a different reaction to streptozotocin (STZ) among different strains [19].

In the last years, diverse information was produced about RGC loss based on these animal models of diabetes. Because of this, the aim of this review is to shed light on the molecular mechanisms behind RGC damage and loss related to diabetic retinopathy. If appropriate, the review is also intended to provide information about studies on molecules tested as possible therapeutic agents for the prevention of RGC loss. Due to the known effects of Alpha-1 antitrypsin (A1AT) on signaling pathways and targets affected by diabetic retinopathy in RGCs and in other cells, tissues or pathologies, we also propose it as a new therapeutic approach to delay, reduce or avoid RGC loss (Table 1).

For this review a literature search was performed using medline/pubmed. The terms searched were (1) retinal ganglion cells and diabetic retinopathy, (2) retinal ganglion cells and diabetes, (3) alpha-1-antitrypsin and diabetes, (4) diabetic retinopathy and alpha-1-antitrypsin, (5) alpha-1-antritrypsin and inflammation.

## 2. RGC Loss in Diabetic Retinopathy

First observations about RGCs in animal models of diabetic retinopathy found impairment of retrograde axonal transport and dysregulation of glutamate release. Although RGC loss is not mentioned in these studies, the events finally observed, are contributors to RGC damage and loss. On the other hand, most recent publications focus on a direct RGC loss. For this reason, this section is divided into indirect and direct contributors to RGC loss.

### 2.1. Indirect Contributors to RGC Loss

#### 2.1.1. Retrograde Axonal Transport Impairment

One of the first experimental observations seen in animal models of diabetic retinopathy was impairment of retrograde axonal transport on RGCs. This impairment was found to be even greater in type 1 than in type 2 diabetes, and possibly due to metabolic dysfunctions. This event may contribute to optic nerve atrophy [20]. It was also observed that the polyol pathway metabolism was involved in this process [21].

One of the current treatments for advanced diabetic retinopathy involves the use of intravitreal injections of anti-vascular endothelial growth factor (VEGF) agents [8,9,10]. Although this treatment is useful to treat the vascular injury, it was observed that VEGF-A antagonists contribute to a distal reduction in the superior colliculus transport and RGC loss in the Ins2(Akita) diabetic and JR5558 spontaneous choroidal neovascularization mice [22].

#### 2.1.2. Glutamate Release

Glutamate is a neuro-transmission factor released by photoreceptors, bipolar cells and RGCs. Glutamate receptors are important for normal visual activation of RGCs [23,24,25].

In an in vitro culture of RGCs under high glucose conditions, an important increase in glutamate release was observed, causing a significant extracellular glutamate accumulation. This accumulation of glutamate also produced neurotoxicity of RGCs, further deteriorating injuries on these cells [26].

### 2.2. Direct Contributors to RGC Loss

Retinal neuropathy on diabetic retinopathy involves progressive RGC death, axonal degeneration and, consequently, optic nerve degeneration [27]. RGC loss occurs in diabetic patients even prior to diabetic retinopathy diagnosis. This loss is enhanced with diabetic retinopathy progression. This RGC damage can be detected in patients using optical coherence tomography (OCT) [28]. Similarly, in experimental diabetes, loss and morphological changes of RGC were seen in animal models. There are some discrepancies in the occurrence of RGC loss probably due to a different reaction to streptozotocin (STZ) among different strains. Anyway, loss of RGC function might occur before morphologic changes [29]. One of these changes is the enlargement of the dendritic field, probably as a compensatory response to the overall loss of RGC [30].

#### 2.2.1. Neurotrophic Factors in RGC Loss

**Figure 1:**
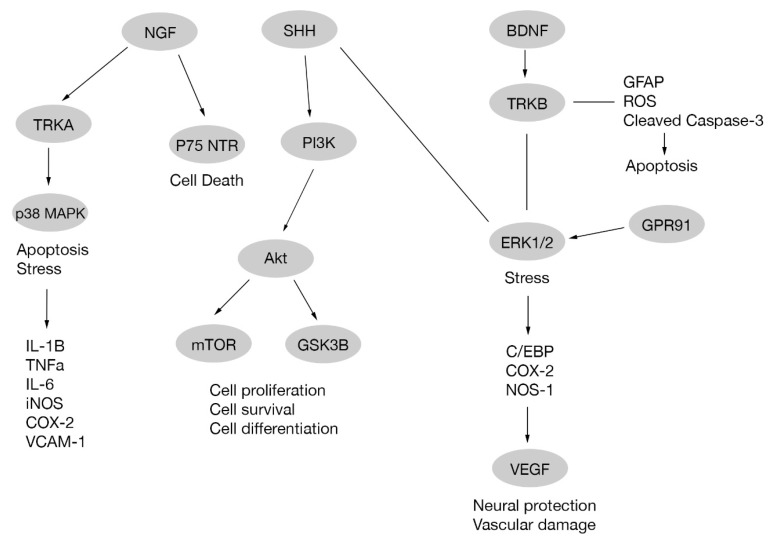
Pathways scheme for neurotrophic factors, mitogen-activated protein kinase cascades and protein kinase B in retinal ganglion cells (RGC) cells.

Brain-derived neurotrophic factor (BNDF) is a protein that enhances insulin activity in diabetic rodents [31,32,33] and promotes neuronal survival [34,35]. In diabetic retinopathy a reduced BNDF expression is observed [27]. Treatment with some compounds, such as erythropoietin in diabetic rats and edaravone in diabetic mice, upregulates BDNF expression, avoiding RGC death. Retinal BDNF upregulation through BDNF/TrkB pathway also reduces glial fibrillary acidic protein (GFAP) expression, phosphorylated ERK ½, cleaved caspase-3 expression and reactive oxygen species production [36,37]. In addition, treatment of diabetic rats with neural stem cells originated from mesenchymal stem cells prevented BDNF decrease, improving leakage, morphological changes and vision, and hindering the progression of diabetic retinopathy [38]. Other treatments involving transplanted CD133+ cells (where CD133 is a pentaspan membrane glycoprotein used as a stem cell biomarker [39]) in STZ-induced diabetic mice retina preserved the histological structure of inner retina, RGCs and rod-on bipolar cells. In addition, CD133+ cells differentiated to RGCs and expressed BDNF promoting the survival of RGCs and other retinal cells [40], Figure 1.

The nerve growth factor (NGF) is a polypeptide member of neurotrophins family, which also includes BDNF [41]. NGF activates two distinct cell surface receptors: tyrosine kinase A receptor (TrkA) related to signaling pathways involved in cell survival, proliferation and differentiation [42,43], and p75 neurotrophin receptor (p75 NTR) [44] involved in neuronal death [45,46]. In diabetes there is an increase in lipid peroxidation and, consequently, nitration of TrkA receptor occurs. This results in an impairment of TrkA expression and phosphorylation leading to a diminished activation of its target Akt. Additionally, there is an increase in the pro-apoptotic p75NTR expression in RGC. Altogether, the effects produced on both receptors lead to RGC death. These effects are reverted with epicatechin [47], a dietary supplement, or exogenous NGF administration [48]. A study performed on diabetic rats shows an increase in NGF on diabetic retina probably due to an endogenous protective response against RGC degeneration, and this effect is enhanced by NGF administration and diminished by anti-NGF agents [49]. Nevertheless, a report suggests that neuronal death occurs despite NGF administration [50] Figure 1.

Ciliary neurotrophic factor (CNTF) is a different neurotrophic factor that has no homology with the other neurotrophic growth factors, BDNF and NGF [51]. In diabetic rat retinas, CNTF mRNA expression is reduced. Using intraocular treatment of CNTF, recovery of RGCs and dopaminergic amacrine cells from degeneration is observed [52].

Another related growth factor recently described, called mesencephalic astrocyte-derived neurotrophic factor (MANF) [53], was also reported to act on RGCs as a protective factor against hypoxia-induced cell injury [54] making this protein an interesting target to be studied in retinal diseases as diabetic retinopathy.

#### 2.2.2. Mitogen-activated Protein Kinase Cascades 

Signals travel along each cell from membrane receptor to cytoplasmatic targets and/or nuclear targets producing different responses upon cell stimulation. In this process specific pathways are activated or inhibited and sometimes a group of mitogen-activated protein kinase (MAPK) cascades are included. MAPK pathway comprises a big variety of proteins including p38 mitogen-activated protein kinase (p38 MAPK), ERK1/2 and c-Jun N-terminal kinase (JNK) cascades. p38 and JNK cascades are part of stress signals, while theERK1/2 cascade is related to mitogenic signals [55].

p38 activation has been observed in response to different physical and chemical stimuli. As a consequence, there is a production of pro-inflammatory cytokines (IL-1β, TNF-α, IL-6); induction of enzymes like COX-2; iNOS; VCAM-1 and other adherent proteins. Activation of p38 is also related with apoptosis and is induced by NGF through TrkA [56].Under high glucose stress produced in in vivo or in vitro diabetes models, there is an activation of p38 MAPK in RGCs. This activation was observed to be diminished with nitrotyrosine [50], Nmnat1 knockdown [57,58], chlorogenic acid, ferulic acid, and rutin present in He-Ying-Qing-Re Formula [59] and with hesperdin, an antioxidant molecule. Another member of the MAPK family is JNK. Similar to p38 MAPK, it is activated under high glucose conditions and also attenuated with hesperidin [60]. In addition, an increased phosphorylation of p38 MAPK and other downstream targets have been associated with RGC death and impairment of axonal transport in retinal neurons after VEGF-A antagonist administration [22].

There are many different pathways that act on the cell indirectly through MAPK. One of them is the Sonic hedgehog signaling pathway (SHH), upregulated in the retina of diabetes rat models and also in Müller cells cultured under high glucose conditions. Exogenous activation of SHH showed neuroprotective effects acting via muller cells downregulating ERK1/2 or activating PI3K pathways without producing changes on p38 MAPK expression [61].

In diabetic retinopathy and retinopathy of prematurity the presence of hypoxia causes upregulation of the expression of G protein-coupled receptor 91 (GPR91) and consequently augmented expression of VEGF through ERK1/2-C/EBP β (c-Fos) and/or ERK1/2-COX-2/PGE2 signaling pathways [62] Figure 1.

#### 2.2.3. Protein Kinase B

Protein kinase B (PKB, or Akt) is a key mediator linking a large number of pathways involved in diverse processes like cell metabolism, proliferation, growth, and survival. Akt activation is coordinated by the protein phosphoinositide-3-kinase (PI3K) [63]. It is already known that the PI3K/Akt pathway is affected in diabetes and inflammatory processes [64,65].

In diabetic retina, there is a downregulation of Akt phosphorylation promoting RGC apoptosis. Increasing Akt activity with drugs like baclofen protects RGCs from cell death [66]. The phosphorylated Akt pathway acts as a neuroprotector through VEGF. For this reason, although anti-VEGF is useful to treat diabetic macular edema and proliferative diabetic retinopathy, it has been demonstrated that VEGF inhibition significantly increases RGC apoptosis and neuronal cell apoptosis in the diabetic retina [67].

Even before RGC apoptosis, synaptic neurodegeneration of RGCs occurs, being one of the earliest events in diabetic retinopathy. A recently identified contributor of RGC synaptic neurodegeneration is hyperphosphorylated-tau (microtubule-associated protein), also associated to other neurogenerative diseases like Alzheimer disease. This hyperphosphorylation of tau is mediated by Akt/GSK3β signaling [68]. Glucagon-like-peptide 1 receptor (GLP-1R) agonists, like liraglutide, can arrest retinal neurodegeneration promoted by hyperphosphorylated tau in a diabetic retinopathy model via activation of GLP-1R/Akt/GSK3β signaling [69]. In addition, compounds like Ginsenoside Rg1 (G-Rg1), an active ingredient in *Panax ginseng* (Asian or Korean ginseng), can avoid hyperphosphorylated tau-induced synaptic neurodegeneration via activation of IRS-1/Akt/GSK3β signaling on RGCs [70], as in Figure 1.

#### 2.2.4. The Nuclear Factor Erythroid 2-related Factor 2

The nuclear factor erythroid 2-related factor 2 (Nrf2) is one of the most important transcription factors involved in cellular detoxifying and antioxidant responses, promoting the expression of diverse cytoprotective genes. Consequently, the Keap1/Nrf2/ARE pathway plays a key role in the maintenance of cellular homeostasis protecting cells under inflammatory, pro-apoptotic and stress conditions [71,72]. The Keap1-Nrf2 system has been related to metabolic and energy balance regulation [73]. Perturbations on the Keap1/Nrf2 system, like in diabetes mellitus, promote the disease condition [74].

It has been observed that enzymes like heme-oxygenase 1 (HO-1) [75], or suppressing long non-coding RNAs like Sox2 overlapping transcript (Sox2-OT) [76], activate the Nrf2/HO-1 pathway protecting RGCs and rodent retinas from oxidative stress, inflammation, and cell apoptosis. The same effect was also observed with different molecules like eriodictyol [77] and lycium barbarum polysaccharides [78].

#### 2.2.5. Nuclear Factor-kappaB

Nuclear factor-kappaB (NFkB) is a nuclear transcription factor that is involved in development of inflammation processes related to diabetic retinopathy and other diseases [79,80]. In some cases, NFkB mediated signaling pathway activation occurs through interaction with HMGB1 and RAGEs, and inhibition of HMGB1 reduce retinal damage induced by high glucose in vitro and in vivo [81,82,83,84]. In RGCs inhibition of HMGB1 inhibits inflammation and promotes RGC survival through the HMGB-1-TLR4-NF-κB signaling pathway [85].

Toll-like receptor 4 (TLR4) and Toll-like receptor 2 (TLR2) are proteins involved in pathogen recognition and innate immunity activation [86]. Silencing TLR4 and TLR2 also promotes a downregulation of NFkB expression. Consequently, it is observed a reduction of secretion of inflammatory molecules like TNF-α and IL-8 promoting RGC survival [87]. In RGCs cultured with high glucose, TLR4 is increased and also its downstream signaling molecules MyD88, NF-κB, TRAF6, NLRP3 and pro-inflammatory cytokines (IL-1β, IL-18). With a treatment with an agonist of TLR4 (TAK-242), inflammation and apoptosis are reduced [88]. 

In animal models hyperglycemia (C57 mice treated with STZ) induces NFkB activation through O-GlcNAcylation of the p65 subunit of NF-κB, promoting RGC death [17]. A downregulation of this process is possible with *Aralia elata* treatment, reducing apoptosis of RGCs and diabetes induced neurodegeneration [89,90].

An induced apoptosis and oxidative stress have been observed due to treatment with hydrogen peroxide (H2O2) in RGC-5 cells (a RGC cell line), activation of NF-κB is produced and inhibited with Formononetin, showing that its activation is not only related to inflammation, but also to reactive oxygen species and apoptosis [91].

**Figure 2:**
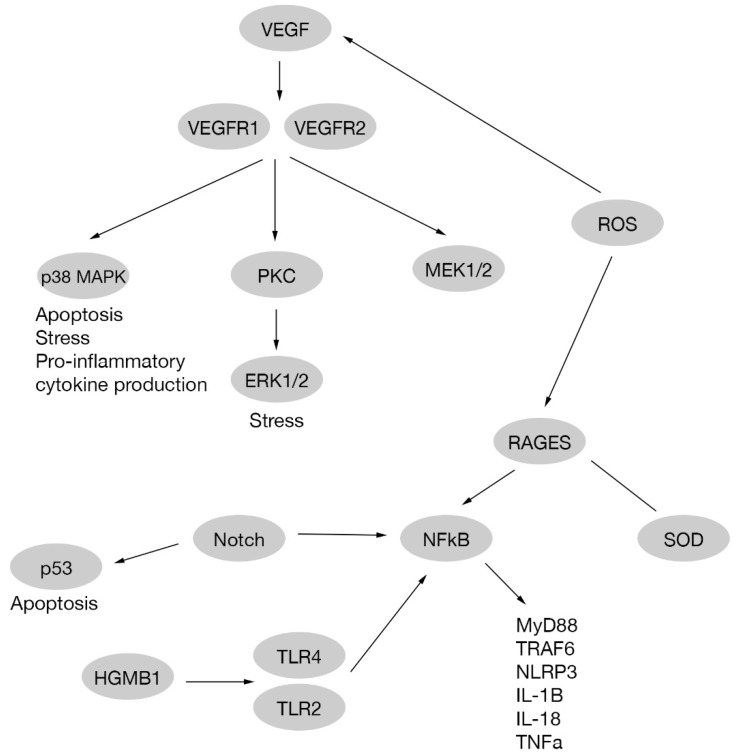
Pathways scheme for vascular endothelial growth factor, mitogen-activated protein kinase cascades, nuclear factor-kappa B and reactive oxygen species in RGC cells.

A study indicates that ERK1/2, like other pathways, is related to NFkB activation. In this research, treatment with an Asian plant extract called astragaloside IV was performed, observing a decrease in ERK1/2 activation, and therefore reducing RGC dysfunction on db/db mice with diabetic retinopathy [92]; Figure 2.

#### 2.2.6. Reactive Oxygen Species

Reactive oxygen species (ROS) are molecules chemically reactive containing oxygen and one extra electron, making them unstable, reactive and oxidant [93]. Although ROS like superoxide anion (O•), hydrogen peroxide (H2O2), and hydroxyl radical (OH−) are naturally formed by mithocondrial electron transport chain, antioxidant molecules are needed to keep the redox balance. Superoxide dismutase (SOD), glutathione peroxidase, catalase, HO-1, glutaredoxins and peroxiredoxins are some antioxidant examples. When the balance is broken by an excess of ROS production, cellular processes are affected [94,95]. Chronic hyperglycemia is involved in ROS production upregulating polyol, the protein kinase C (PKC) and hexosamine pathways [96]. An increase in ROS production by high glucose levels is involved in apoptosis and VEGF production. In addition, ROS promotes a decrease in SOD and other antioxidant molecule activities through AGE receptors (RAGEs) [97,98] Figure 2. 

In RCGs it was observed that crude saponins of *P. notoginseng* and He-Ying-Qing-Re Formula inhibited apoptosis and suppressed ROS by eIF2α/ATF4/CHOP and caspase 12 pathways in in vitro experiments [59,99].

In animal models of diabetic retinopathy, a decreased activity and mRNA of MnSOD are related to apoptosis of retinal neurons including RGCs. Astaxanthin, a carotenoid, reduced apoptosis of RGCs improving oxidative stress markers like superoxide anion, malondialdehyde, 8-hydroxy-2-deoxyguanosine and MnSOD activity. In addition, astaxanthin attenuated hydrogen peroxide(H2O2)-induced apoptosis in vitro (RGC-5 cells) [100,101]. Besides, in vitro incubation of explanted retinas from 12- and 20- week db/db mice with SOD protects RGC functions [102].

#### 2.2.7. Vascular Endothelial Growth Factor

Vascular endothelial growth factor (VEGF) is an important signaling molecule involved in increased vascular permeability and angiogenesis. VEGF plays a neuroprotective role in early phases of diabetic retinopathy when it is released as a consequence of neuronal damage. An example is previously mentioned in Section 2.2.3, where VEGF inhibition increased RGCs apoptosis and neuronal damage in diabetic retinopathy through Akt phosphorylation [67]. Further, as mentioned in Section 2.2.1, different mice models of diabetes presented an impairment of axonal transport after anti-VEGF administration with a phosphorylation of p38 MAPK [22]. Furthermore, VEGF may increase VEGF production, through ERK1/2 [62] or as a consequence of ROS production [98].

Nevertheless, VEGF promotes vascular changes that contribute to diabetic retinopathy progression [103]. Because of this, intravitreal injection of anti-VEGF agents is a natural option to treat diabetic macular edema and proliferative diabetic retinopathy [8,9,10], as well as other ocular like diseases as exudative aged-related macular degeneration [104]. Ranibizumab (Lucentis^®^; Genentech, Inc., South San Francisco, CA), a humanised monoclonal antibody antigen-binding fragment [105], and Aflibercept (Eylea^®^; Regeneron Pharmaceuticals, Inc., Tarrytown, NY), a soluble decoy receptor fusion protein [106], are indicated for the treatment of patients with diabetic retinopathy. Bevacizumab (Avastin^®^; Genentech, Inc., South San Francisco, CA) it is a recombinant monoclonal anti-VEGF antibody that was approved as an antiangiogenic agent to treat cancer, and is used off-label to treat different neovascular ocular diseases [107]. 

A study carried out in streptozotocin induced diabetic retinopathy in rats showed reduced vascular angiogenesis and damage with intravitreal injections of Ranibizumab in early stages of diabetes. Moreover, in the same study, it was observed that RGC numbers are preserved with this anti-VEGF agent [108].

In the same animal model, a decrease in VEGF expression was observed in retinal vessels of diabetic rats treated with Icariin, a flavonoid molecule. In the same research, Icariin produced an enhanced neurite growth in RGCs [109]. This result is unexpected and needs to be confirmed by other investigators because increase VEGF expression is considered neuroprotective. Perhaps, Icarin protects RGCs through an unknown mechanism. 

Endogenous VEGF-A165a and VEGF-A165b are two alternative RNA splicing of VEGF molecule. Particularly, VEGF-A165b is related to neuroprotective effects on neurons. Therefore, a recombinant human VEGF-A165b was developed and tested in rats with retinal damage by ischemia-reperfusion, observing a protective effect on RGCs through activation of VEGFR2, MEK1/2, and inhibition of caspase-3 [110].

VEGF receptors (VEGFR1, VEGFR2) are also related to different signaling pathways in high glucose treated human endothelial cells. These pathways are p38MAPK/STAT1 and PKC-Erk1/2-NOS1, that cause apoptosis and vascular hyperpermeability, respectively [111]. A study on RGCs reports that Nipradilol (a beta-blocker and nitric oxide donor) prevents apoptosis through the nitric oxide pathway in vivo and in vitro [112]; Figure 2.

#### 2.2.8. Other Inflammatory Molecules

As a consequence of high glucose levels, glucose-mediated microvascular damage, cellular oxidative stress, protein kinase C activation and superoxide overproduction emerge, and later, a chronic inflammation state is established. Inflammation in the retina triggers microglia activation [113,114,115], ROS production [116,117], enhancement of NFkB and ERK activation [115] and, finally, production of pro-inflammatory cytokines, such as tumor necrosis factor (TNF-α), interleukins (IL)-1β, IL-6, IL-8, vascular cell adhesion molecule (VCAM-1) and intracellular adhesion molecule 1 (ICAM-1) [118,119]. 

Cytokines production and the presence of other kind of molecules, modify drastically the environment in the retina, affecting neuronal cells being the first cells damaged. The relationship between vascular damage and neuronal damage is less clear, and two theories have been proposed. First hypothesis is that neuronal damage and vascular damage are independent events, but seems not probably because of the deep connection between neuronal, glial and vascular cells in the retina. This leads to a second hypothesis, where VEGF produced on early stages of the disease to protect neuronal cells, also causes angiogenesis and vascular damage at late stages. Moreover, same molecules that triggers neuronal damage in the retina, like glutamate, also increase VEGF production [103] Figure 2.

In RGCs co-cultured with Müller glia, it was observed that addition of pro-inflammatory cytokines has a direct negative effect on RGC survival. Moreover, with high glucose media culture conditions, TNF-α, IL-1β and IL-6 were augmented, and normalized with dexamethasone (an anti-inflammatory agent), indicating that anti-inflammatory strategies are useful to promote RGC resilience [120]. 

In diabetic db/db mice where increased RGC apoptosis is normally observed, expression of microglia markers like IBA-1 and F4/80 are also augmented [121].

Transfection of miR-145-5p inhibitor (inhibits fibroblast growth factor 5, FGF5) in RGC-5 cells exposed to high glucose, as in an in vitro diabetic retinopathy model, produces a decrease in TNF-α and IL-6 on these cells and also reduces apoptosis, keeping an elevated cell proliferation capacity [122]. 

In a research study carried out in diabetic mice, RGC loss was produced by matrix metalloproteinase 9 (MMP-9) [123], a modulator of inflammation [124]. In addition, production of advanced glycation end-products (AGEs) was related to neuronal dysfunction in diabetic retinas [125]. Furthermore, a research study carried out in diabetic rats on and without a high-fat-diet (HFD) reports a higher percentage of ganglion cells stained with RAGE in diabetic animals on HFD compared to diabetics fed on a normal diet [126].

#### 2.2.9. Other Apoptosis and Autophagy Related Pathways

The Notch pathway plays an important role in cell fate determination and also interacts with different pathways related to apoptosis such as p53, NF-κB and PI3K/Akt pathways [127]. In an in vitro RGC culture under high glucose conditions, cell death produced by glucose was reduced via Notch signaling activation, indicating that Notch could be a potential target to preserve RGCs in diabetic retinopathy [128,129]; Figure 2.

The pro-apoptotic RNA-dependent protein kinase (PKR) signaling pathway is involved in diverse cellular processes like mRNA transcription and translation, apoptosis and proliferation. Dysregulation on this pathway has been associated with metabolic disorders and inflammation [130,131,132]. In a STZ-induced diabetes model, upregulation of miRNA miR-29b protected RGCs from apoptosis by PKR signaling pathway [133].

mTOR is a key regulator of different cell activities like cell growth and autophagy [134]. In diabetic retinas an evident downregulation of phosphorylation of mTOR was observed; thus, RGC apoptosis increased. With an autophagy inhibitor (3-MA), decreased RGC death was observed [135].

#### 2.2.10. Other Treatments in Different Diabetic Animal Models with RGC Loss

In streptozotocin induced diabetes, retinal injury like vasculature damage, RGC loss and a consequently visual pathway dysfunction can be observed [136]. With resveratrol treatment, a polyphenol with antioxidant properties [137], RGC loss is prevented through calcium-calmodulin dependent protein kinase II downregulation [138]. In addition, the leukemia inhibitory factor, a molecule previously related with neuroprotective properties in retinal disease [139], protects retinal vasculature and RGCs in diabetic animals [140].

In a study carried out in a different diabetes rat (the Otsuka Long–Evans Tokushima fatty model), effects of ciclostazol were tested. Ciclostazol is a phosphodiesterase-3 inhibitor with anti-platelet, anti-inflammatory, and vasodilatory effects [141]. In these diabetic rats, an increase in the retinal RGC layer was observed after treatment [142].

In different diabetes mouse model, Ins2Akita mice, serine racemase was associated with neuronal degeneration. For this reason, retinas from Ins2Akita-Srr mice with less D-Serine proteins were tested, where less RGC loss was observed indicating D-Serine as an important factor for RGC degeneration [143]. In other transgenic mouse model, Sigma receptor 1 knock out subjected to diabetes chronic stress, RGC dysfunction was also observed [144]. 

### 2.3. Diabetic Retinopathy as a Risk Factor for Other Ocular Diseases that also affect RGCs

RGCs, one of the most important cell types in the retina, are affected in other diseases not necessarily related with diabetic retinopathy. When these diseases occur in a diabetes context, diabetes could work as an ‘exacerbating risk factor’ for these diseases.

In open-angle glaucoma, there is progressive apoptosis of RGCs and optic disk excavation. In animal models of diabetic retinopathy and open-angle glaucoma, there is increased apoptosis of RGCs [145].

In hyperhomocysteinemia, a medical condition characterized by an abnormally high level of plasmatic homocysteine, RGC loss is present. When hyperhomocysteinemia manifests with diabetic retinopathy, RGC loss is even more elevated in animal models [146].

Wolfram syndrome 1 (WFS1, OMIM 222300), an uncommon genetic disorder caused by mutations in WFS1 gene (wolframin protein), is accompanied by optic nerve atrophy, deafness, diabetes insipidus and diabetes mellitus. In RGCs, there is loss of wolframin expression, possibly producing the optic nerve atrophy observed in this disease [147].

Diabetic retinopathy also affects the development and progression of ocular hypertension and glaucoma through loss of RGCs in diabetic patients. In contrast, in a D2.Ins2Akita/+ mouse model with increased intraocular pressure and vascular leakage, RGCs do not lose axon transport or degenerate [148].

### 2.4. Contribution to Circadian Activity

Melanopsin is a key regulator of the circadian clock and is expressed by RGCs. Diabetic retinopathy affects RGCs that express melanopsin disturbing circadian activity. As a consequence, patients with diabetic retinopathy may show altered blood pressure, sleep disorders and other related circadian activities [149]. Furthermore, melatonin, other molecule involved in circadian physiology, has been related to anti-inflammatory effects in ocular diseases [150]. Considering this collectively, it is clear that RGC involvement plays an important role in diabetic retinopathy pathophysiology. 

## 3. Possible Role of Alpha 1 Anti-trypsin in RGC Damage and Loss

In the complex environment context generated by diabetic retinopathy, multiple signaling pathways and processes are involved in RGC damage. Although promising therapies are being developed for the prevention of RGC loss, it is evident that a multifactorial approach targeting a wide range of signaling pathways and inflammatory molecules that promote retinal damage is needed (Table 1).

**Table 1:** Mechanisms involved in RGC loss in diabetic retinopathy and A1AT possible role.

Name	Molecule	Targets or Molecules Produced	Produces	Diabetic Retinopahty	A1AT Possible Effect	References
Glutamate	Glutamate		Citotoxicity	Increased	Decrease	[26,151,152]
Neurotriphins	BDNF	TrkB	Protection	Decreased	Increase through TrkB	[27,36,37,38,39,40,153]
	NGF	TrkA/Akt	Protection	Decreased	Increase through TrkA	[42,43,45,46,47,153]
		p75 NTR	Neuronal damage	Increased		
	CNTF	CNTF mRNA	Protection	Decreased		[52]
	MANF		Protection			[54]
MAPK	p38	pro-inflammatory cytokines	Inflammation	Increased	Decrease	
		Apoptosis	Cell loss			[22,50,56,57,58,59,154]
		Adherent proteins expression	Inflammation			
	JNK	NFkB	Inflammation	Increased	Decrease	[60,155,156,157]
	ERK	VEGF	Vascular angiogenesis	Increased	Decrease VEGF through VEGFR1, VGFR2/p38 MAPK/STAT1	
			Apoptosis			[62,108,109,158,159]
			Neuronal damage			
		NOS1	Apoptosis	Increased	Decrease NOS1 through PKC/ERK	[56,160]
			Hyperpermeability			
	GPR91	VEGF	Vascular angiogenesis	Increased	Decrease through VEGFR1, VGFR2/p38 MAPK/STAT1	
			Apoptosis			[62,158,159]
			Neuronal damage			
Akt	Akt	PI3K/Akt/GSK3β	Inflammation	Increased	Decrease	[66,161,162]
		IRS-1/PI3K/Akt	Inflammation	Increased	Decrease	[70,161,162]
		GLP-1R	Protection			[69]
		Akt/mTOR	Protection	Decreased	mTOR modulation	[135,163,164]
		Notch/PI3K/Akt	Apoptosis	Increased	Decrease	[128,129,161,162]
Nrf2	Nrf2	Nrf2/HO-1	Protection	Decreased		[75]
		SOX2-OT/Nrf2/HO-1	Protection	Decreased		[76]
NFkB	NFkB	NFkB/HMGB1	Inflammation	Increased	Decrease	[81,82,83,84,85,87,88,92,165,166]
		NFkB/RAGEs				
		NFkB/TLR				
		NFkB/ERK				
ROS	ROS	SOD	Protection	Decreased	Increase through RAGEs	[97,98,102,154,158]
		Glutation peroxidase	Protection	Decreased		[94,95]
		HO-1	Protection	Decreased	Possible modulation	[94,95]
MMP	MMP9		Inflammation	Increased	Decrease	[123,158]
			Cell loss			
AGEs	AGEs		Neuronal damage	Increased		[125,126]
p53	p53		Apoptosis	Increased	Decrease	[128,129,167]

Alpha-1antitrypsin (A1AT) is a sialoglycoprotein of 52 kDa, encoded by the gene SERPINA1 [168]. It is produced as an acute phase protein by hepatocytes, but it is also produced in lungs, alveolar macrophages and neutrophils [169]. A1AT works like a protease inhibitor of different proteins related to inflammation processes, such as proteinase-1, elastase, thrombin and trypsin [169,170]. During a disease or in response to inflammation or tissue injury serum concentrations of A1AT change [171]. A1AT is currently used to treat chronic obstructive pulmonary disease and A1AT deficiency [172,173]. Recently, A1AT has been proposed as a possible therapeutic approach for diabetic retinopathy based on its anti-inflammatory effects. In fact, A1AT is a molecule involved in several mechanisms observed in DR, such as anti-inflammatory processes, avoidance of apoptosis and extracellular matrix remodeling, as well as protection of vessel walls and capillaries [174]. Furthermore, our group tested A1AT in a type 1 diabetes mouse model (streptozotocin model) and observed a reduction of inflammation and retinal neurodegeneration [175]. Furthermore, in a recent publication made by our group, we elucidated some of the molecular mechanisms behind A1AT treatment in an in vitro study of RPE cells exposed to high glucose. Some of the pathways modulated by A1AT treatment are altered with diabetic retinopathy affecting RGCs, like mTOR, Akt and NFkB [176]. It is worth mentioned that these pathways modulated by A1AT in RPE cells are in also in accordance with results of investigations carried out in non-ocular tissues which are described in the following paragraphs.

### 3.1. A1AT and Glutamate Release

As previously mentioned, an increase in glutamate release by RGCs occurs in diabetic retinopathy. Excessive glutamate accumulation produces RGC toxicity. It has been demonstrated that A1AT prevents glutamate toxicity in neurogenerative models like Alzheimer’s disease, where cytotoxicity triggers neurodegeneration [151,152].

### 3.2. A1AT and Mechanisms Associated with Inflammation and RGC Loss

#### 3.2.1. A1AT and Neurotrophic Factors

ERK is increased in RGCs under diabetic retinopathy conditions, promoting expression of inflammatory molecules. BDNF, GNDF and CNTF produce an anti-inflammatory effect which decreases ERK signaling through TRKs. Although it has not been demonstrated that A1AT could interact with TRKs, it can modulate ERK signaling. Native form of A1AT in airway-derived cells promotes anti-inflammatory signaling with a reduction in ERK and NFkB signaling through EGF receptor and ADAM17 activity [153]. In addition, a mutant form of A1AT promotes pro-inflammatory phenotype in chronic obstructive pulmonary disease [177].

#### 3.2.2. Mitogen-Activated Protein Kinase Cascades 

It has been demonstrated than A1AT reduces p38 MAPK activity, as well as STAT1, H2O2, ROS generation and inflammatory cytokines in preeclampsia models. A1AT also increases SOD activity in the same model, suppressing oxidative stress [154]. Moreover, A1AT reduces JNK activation and consequently NFkB activation in β-cells grafts, increasing cell survival [155,156,157]. Reducing p38 MAPK and JNK activity could be useful in RGCs where their activity is increased in the presence of diabetic retinopathy. 

#### 3.2.3. Protein Kinase B

In diabetic retinopathy there is a downregulation of Akt activity affecting a large number of pathways related to diverse cellular processes; ultimately, the resulting imbalance promotes RGC apoptosis. In the literature there are reports stating that A1AT can downregulate the PI3K/Akt/mTOR pathway in breast cancer cells, where this pathway is increased [163,164]. However, there are other reports remarking the role of A1AT as an up-regulator of the PI3K/Akt pathway, reducing neutrophil elastase-induced migration of lung cancer cells, tumor tissue proliferation and inflammatory microenvironment [161,162]. Furthermore, A1AT was demonstrated to produce an anti-apoptotic effect avoiding PKC/Akt inactivation in neutrophils from patients with systemic inflammatory response syndrome [160]. Also, loss of A1AT expression is related with epithelial mesenchymal transition (EMT) in hepatocellular carcinoma [178].

#### 3.2.4. The Nuclear Factor Erythroid 2-related Factor 2

Nrf2 and HO-1 are molecules involved in the protection of RGCs in an inflammatory environment. Interestingly, there is dysregulation of Nrf2 in A1AT deficiency by NFkB signaling, producing tissue remodeling, fibrosis and alveolar damage [179]. In Alzheimer disease, HO-1 is increased and contributes to derangement, and A1AT was observed to be a HO-1 suppressor in brain, plasma and peripheral tissues [180].

#### 3.2.5. Effect of A1AT on Reactive Oxygen Species and Inflammatory Agents

Oxidative stress and inflammation are major contributors to RGC loss. A1AT exhibited an antioxidant role in preeclampsia by reducing ROS and MMP9 and increasing SOD through inactivation of STAT1/p38 signaling [158]. 

A1AT also inhibits MMP9 activity in liver cells under hypothermia and hypoxia-reoxygenation injury, reducing nitric oxide production and protecting them from apoptosis [181]. However, in blood neutrophils cultures, A1AT treatment increased MMP9 release [182]. Furthermore, A1AT suppressed MMP12 and TNF-α in stimulated lung macrophages [183]. 

Our group also demonstrated a reduction in systemic and retinal TNF-α in A1AT treated C57 mice in a streptozotocin induced DR model. Moreover, less retinal neurodegenerative changes were observed [175].

TLR3, TLR4, TLR7 and TLR8 are downregulated by A1AT; consequently, IL-1β induced by TLR4 is also reduced in monocytes and dendritic cells of diabetic patients [184,185].

A1AT decreases NFkB innate immunity related genes IL-6 and IL-8 in aging models [165] and NFkB transcription and translocation in mouse models of acute inflammation [166]. Reduced expression of mRNA of molecules like inhibitor of NFkB (IkB) and ICAM-1 is produced by A1AT [166,186].

In lung cells A1AT suppresses caspase-3 activation and prevents apoptosis by involving a mechanism related to VEGFR1 and VEGFR2 [159]. There is a correlation between mutant p53 (a tumor suppressor protein) and A1AT which suggests A1AT as a target of p53 [167].

## 4. Conclusions

It is evident that there are multiple, complex and interrelated mechanisms behind RGC function impairment and RGC loss in diabetic retinopathy. These include glutamate accumulation and toxicity, reduced expression of neurotrophic factors, signaling pathway impairment and increased production of pro-inflammatory factors. Besides, diabetic retinopathy becomes an exacerbating risk factor when it occurs concomitantly with other diseases affecting RGCs. There are diverse experimental treatments to avoid RGC loss in diabetic retinopathy mentioned in this work; nevertheless, to our knowledge, they are not clinically effective at present. The literature review on A1AT carried out in this work shows A1AT as a potential therapeutic agent for targeting a wide range of mechanisms involved in RGC loss.

## Abbreviations

A1AT	Alpha-1 antitrypsin
AGEs	Advanced glycation end-products
Akt /PkB	Protein kinase B
BDNF	Brain-derived neurotrophic factor
CNTF	Ciliary neurotrophic factor
GFAP	Glial fibrillary acidic protein
HO-1	Heme-oxygenase 1
ICAM-1	Intracellular adhesion molecule 1
JNK	c-Jun N-terminal kinase
MANF	Mesencephalic astrocyte-derived neurotrophic factor
MAPK	Mitogen-activated protein kinase
MMP-9	Metalloproteinase 9
NFkB	Nuclear factor-kappaB
Nrf2	Nuclear factor erythroid 2-related factor 2
NGF	Nerve growth factor
P75 NTR	p75 neurotrophin receptor
PI3K	Phosphoinositide-3-kinase
PKC	Protein kinase C
PKR	Pro-apoptotic RNA-dependent protein kinase
PVR	Proliferative vitreous retinopathy
RGCs	Retinal ganglion cells
ROS	Reactive oxygen species
SHH	Sonic hedgehog signaling pathway
SOD	Superoxide dismutase
STZ	Streptozotocin
TrkA	Tyrosine kinase A receptor
VCAM-1	Vascular cell adhesion molecule
VEGF	Vascular endothelial growth factor
TLR4	Toll-like receptor 4
